# Robust Memristor Networks for Neuromorphic Computation Applications

**DOI:** 10.3390/ma12213573

**Published:** 2019-10-31

**Authors:** Dániel Hajtó, Ádám Rák, György Cserey

**Affiliations:** 1 Faculty of Information Technology and Bionics, Pázmány Péter Catholic University, 1083 Budapest, Hungary; cserey.gyorgy@itk.ppke.hu; 2 StreamNovation Ltd., 1083 Budapest, Hungary; streamnovation@streamnovation.com

**Keywords:** memristor, neuromorphic computing, artificial intelligence, hardware-based deep learning ICs, circuit design

## Abstract

One of the main obstacles for memristors to become commonly used in electrical engineering and in the field of artificial intelligence is the unreliability of physical implementations. A non-uniform range of resistance, low mass-production yield and high fault probability during operation are disadvantages of the current memristor technologies. In this article, the authors offer a solution for these problems with a circuit design, which consists of many memristors with a high operational variance that can form a more robust single memristor. The proposition is confirmed by physical device measurements, by gaining similar results as in previous simulations. These results can lead to more stable devices, which are a necessity for neuromorphic computation, artificial intelligence and neural network applications.

## 1. Introduction

Since the theoretical [1] and practical [2] discovery of memristors, they have been extensively studied [3,4,5] as elementary building blocks for artificial intelligence and neuromorphic computing applications.

The expected properties of memristors for such applications are wide and analog resistance range, low variance of device parameters and high device stability during long-term operation. Research has been done [6] to find optimal materials that satisfy these expectations, but even then there are other possibilities to further increase the capabilities of memristors.

In binary memory applications, three important properties should be considered. The first one is having two clearly distinguishable states and these state declarations should apply to every element in a memory array. The second one is having a fast switching speed between the states. To reach the performance of the current complementary metal–oxide–semiconductor (CMOS) technology’s RAM the switching speed should be less than 10 ns. The third one is cycle endurance, which is the number of write–erase cycles without permanent device failure.

In crossbar-network applications, a certain amount of uniformity of the memristors is necessary. The programming voltage and current levels are the same for every element and thus one expects that they will behave similarly for the same input signals.

In the case of ANN applications, more deviance could be tolerated, but many state devices are needed, so the memristors developed for binary or multi-state memory purposes will not be sufficient.

The mass production of devices, which can reliably fulfill these requirements, is not trivial. If the production yield of single devices is less than 100 percent (as they are not functioning as memristors or they are outside of the accepted range of parameters), then they can also affect the access circuit and the encompassing parts of the neuromorphic system.

If the production yield of single devices is less than 100 percent (as they are not functioning as memristors or they are outside of the accepted range of parameters), then they can also affect the access circuit and the encompassing parts of the neuromorphic system.

In very large scale integration (VLSI) device manufacturing, it is often easier and tends to cause fewer faults to make the same device many times, and use it as a building block to emulate other devices, instead of creating fewer, but different devices [7]. The same approach can be applied to memristors, but one should take into consideration their special nonlinear behavior in the voltage–current domain. This idea is further supported by the fact that memristors as two-terminals, could be manufactured more easily on many layers on microchips [8] than transistors. However, with every extra layer, the probability of device defects could also increase.

In order to maintain or even improve the virtual yield of the production, interconnected structures of the memristor network are proposed. These circuits and the presented measurement results provide a response to the above mentioned challenges. Our proposed circuit constructions can be efficiently implemented on microchips, stacking the memristors of the circuit on top of each other. If a decent multilayer production technology arises with memristors, the disadvantage of the usage of several layers for the implementation of a single layer of memristor would be neglectable.

This paper is organized as follows: after the above problem proposal, the measurement environment is introduced and explanatory discussion is given about our circuitry. The third section contains the proposed circuits and the measurement results that are more detrimental to the yield. This circuitry effectively addresses the proposed task. In the fourth section, the results are summarized and analyzed. The article is closed with a brief summary of the results in the conclusion section.

## 2. Materials and Methods

### 2.1. Materials

The measured memristor devices are made of $Ge_{2}Se_{3}$ (germanium-selenide) and Ag (silver) based chalcogenide dielectric with *W* (Tungsten) conductors. The devices have a switching threshold, meaning that under a certain threshold voltage (0.1 V in our case), their state does not change. This feature makes the memristor implementation desirable for applications where reading the state should not change the state itself. On the other hand, usually it has very few metallic dendrites, which makes the characteristic very coarse. The memristors are current-controlled and the typical writing-erasing voltages are 2.5 V. One of the consequences of being current-controlled is that the erasing process is faster than the writing process.

**Figure 1:**
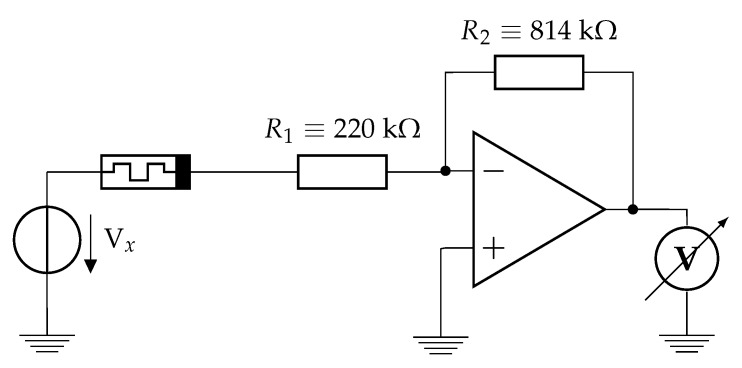
Measurement environment circuit. The measurement setup consists an amplifier circuit as a current controlled voltage source and a current regulator resistor. The used amplifier is a “TL082”. The applied voltage $V_{x}$ was strictly between −2.5 V and 2.5 V. The memristor symbol represents either a single memristor or a network of memristors depending on the measurement.

The measurement setup consists of an amplifier circuit as a current–voltage converter and a current regulator resistor as it can be seen in Figure 1. The current regulator resistor helped to ensure that the current does not reach high values where the device could become faulty. The used signal generator and measurement device is an “NI ELVIS II+”, controlled by LabView software (National Instruments, Austin, TX, United States). The sampling frequency is 500 kSample/s for every measurement. The state of every device has been set to an OFF state before every measurement.

### 2.2. Methods

#### 2.2.1. Metrics

First of all, it is important to differentiate two main types of memristors from a functional point of view. The first type is the analog purpose memristor (APM). It operates in the continuous domain, which means it can have any resistance (or conductance) value in its operational range. This might sound unrealistic as we know that at a very low scale, energy levels are quantized, but it can be interpreted as the memristor having so many states that can be considered as infinitely many. Another formal definition is that an APM can store any real value between the normalized range of zero and one.

The second type is the digital (or discrete) purpose memristor (DPM), which has several but countable states and the resistance value can only be one of these states. An important property is that these states should be clearly distinguishable from each other. This type can be used trivially as an *n* state memory unit based on the number of its possible states.

An extreme, but important case of the DPM is when only two states can be clearly distinguished, as they can be further classified as binary purpose memristors (BPM). With its reduced capabilities they lack applications beside their use as binary memory units supplementary to the CMOS based digital systems or implementing routing in logic gate arrays, like Field programmable gate arrays (FPGAs) [9].

In general, the mass production of BPMs is solved, there are manufacturers [10], who sell commercial devices for an affordable price. DPMs are existing in an early development state at research institutes [11]. APMs, which have practically an infinitely many numbers of states, are yet to be introduced and might even be impossible to produce due to physical limitations [12]; or it requires new quantum mechanical solutions, which are also under development [13]. In general, from an application point of view, digital memory technologies use BPMs, artificial neural networks need at least DPM complexity, and neuromorphic computation applications require APMs.

Our previously introduced circuit proposals [14] were intended to convert several DPMs into a single APM. This was tested through simulations, which showed that this circuit topology can achieve analog behavior when made from solely multi-state memristors. However, in this work real device measurement results are given, which proves that the same circuit can effectively convert several unreliable BPMs into a more reliable one.

The same control signal should produce the same result, both in the transient characteristics and the final state of the memristor. By reliability, we mean a low variance of the characteristics. Our aim was to avoid using a memristor model as an absolute reference, and be able to approximate the real memristors more accurately. Therefore, our analysis focuses on the mean and variance of characteristics of several measurements on the same device or network in a short period of time.

The index of dispersion has been used as the measure of unreliability. It formulates as the sum of the variance of the signal, normalized by the amplitude of the signal, due to the expectation that higher amplitude signals have naturally higher variance. This measure is valid only for positive data points. For this reason, the absolute value of the signal has been used:

(1) $$u=\sum_{i}^{N}\frac{\sigma_{i}^{2}}{|\mu_{i}|}\;,$$

where *u* is the unreliability of the device, *N* is the number of measurement points, *i* is a measurement point of the measuring signal, $\sigma_{i}^{2}$ is the variance of a measurement point over the consecutive measurements and $\mu_{i}$ is the mean of a measurement point over the consecutive measurements.

The approximation of the yield of a production technology is highly dependent on the available number of samples of the given device. having a limited number of devices, this question can not be addressed, but it has been shown in a previous work [14] that the yield of a production technology can be increased with this method.

The planning and execution of the measurements have been carried out with consideration of previous related studies [15] on memristor measuring techniques.

#### 2.2.2. Circuits

**Figure 2:**
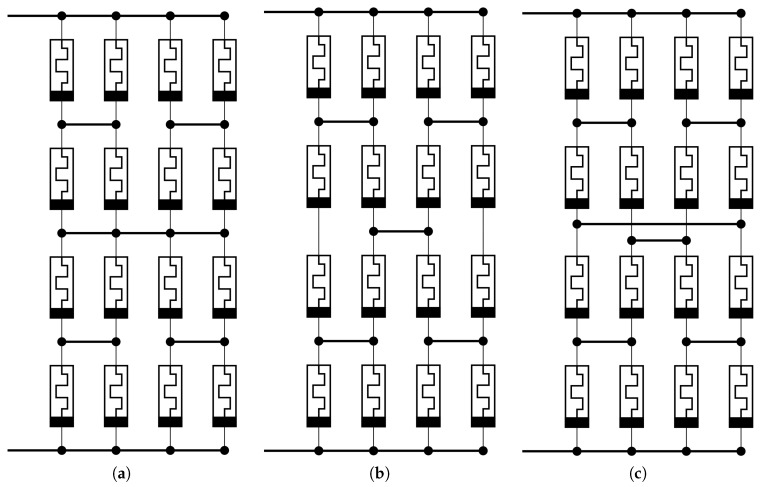
Measured general circuits. (**a**) H-fractal type of array. (**b**) Checkerboard type of array. (**c**) Our newly introduced array.

The measurements were carried out on four different memristor network circuit topologies of which two were introduced before [14] with corresponding simulation results. The H-fractal (Figure 2a) and checkerboard-like (Figure 2b) topology both gave comparably good results, which shows that verifying both cases with measurements is reasonable. During simulations with heavy defect probability, the checkerboard-like topology has given slightly better results.

**Figure 3:**
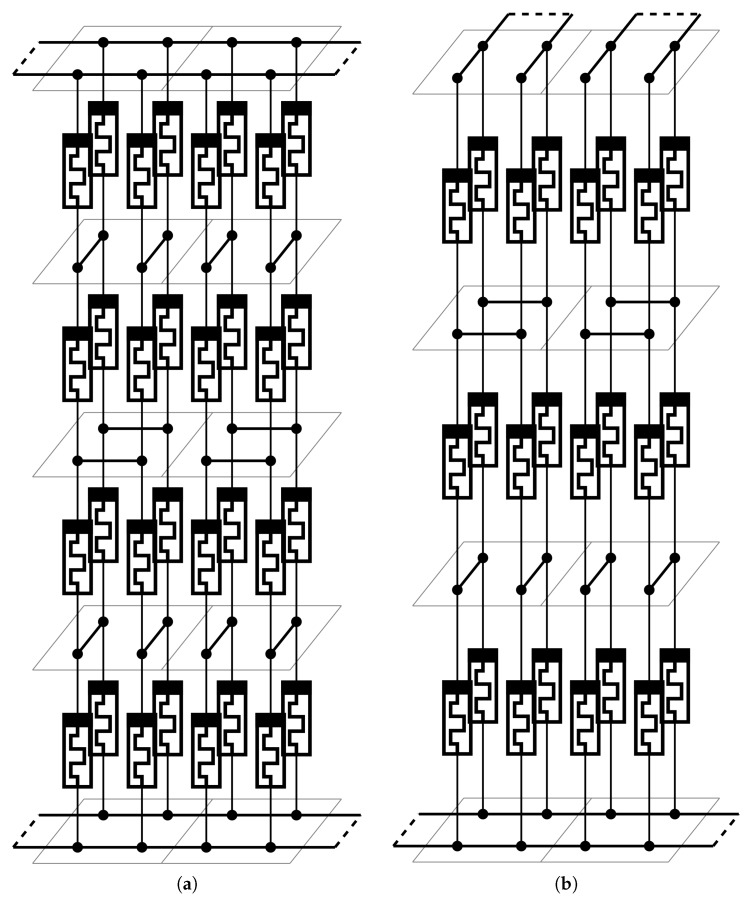
The proposed three dimensional cell structures. (**a**) Two emulated cells from a 2×2×4 array. (**b**) Two emulated cells from a 2×2×3 array.

In this article a third general circuit design is proposed (Figure 2c), which can be implemented as a 2×2×4, three-dimensional grid structure on a multilayer carrier. A structure proposal can be seen in Figure 3a. This new circuit had a better compromise between open and short connection faults, but can only be constructed effectively in a three dimensional structure. The disadvantage is that since the height of the grid was even, and the top and bottom electrodes are aligned, they cannot form a crossbar network.

A workaround could be that this type of network can scale with the height of the 2×2 column, and it can be 2×2×3 or 2×2×5 sized. These new non-general networks result in different memristor parameters. The advantage of an odd height is that it can be realized in a crossbar network as it can be seen in Figure 3b.

Memristor networks that use binary memristors as building components will technically result in a discrete memory capacity as either component can be in the OFF or ON state. The overall resistance value can be calculated for every combination, which is a limited number of possible resistances. However, with sufficiently large grids, this effect can be neglected as the individual operational variances of the elements are also summing up, resulting in a complex macro-characteristics.

Another important property to consider is the used chip area. These networks should be implemented efficiently on a chip as a two dimensional crossbar network. The implementation of the previous networks was only possible using sixteen times more chip area for the emulation of a single device. The new network uses only four times more area with a similar reliability gain, as compared to a single memristor.

## 3. Results

### 3.1. Single Memristor Measurements

**Figure 4:**
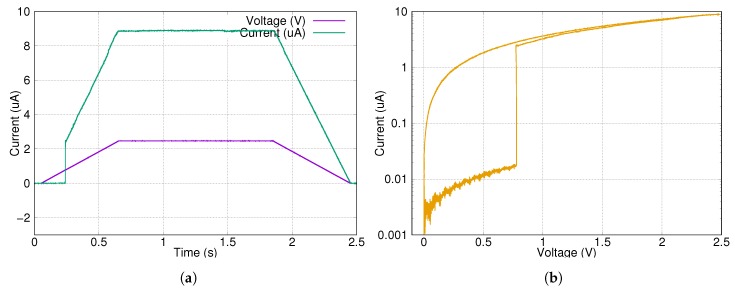
Long timescale measurement on a single memristor device with focus on the writing characteristics. (**a**) The input signal and output response in the time domain. (**b**) Phase portrait of the measurement. Switching is very sharp and the ON/OFF ratio is at least 100.

First test measurements were prepared with a single memristor device. Here two types of signals were used. The first one was a single, 2.4 s long 2.5 V writing pulse, which shows some parameters of the device. The results can be seen in Figure 4. The average ON state was 57 kΩ, the average OFF state was 11.5 MΩ. The ON/OFF ratio is approximately 200.

**Figure 5:**
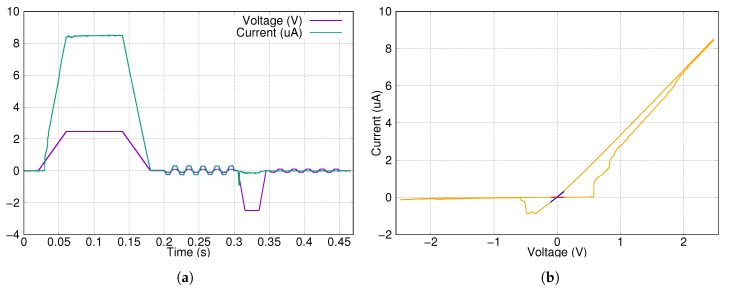
Write-read-erase-read cycle measurement on a single memristor device. (**a**) The input signal and output response in the time domain. (**b**) Phase portrait of the measurement. The read sequence after the write and erase pulses are colored as blue and red, respectively.

The second type of signal is a sequence of a writing and an erasing signal. The writing pulse was 160 ms long, while the erasing one was shorter, 40 ms. The results can be seen in Figure 5. The writing process was faster and starts at a lower voltage level, but the switching was not as sharp as in the previous case (Figure 4). During the reading sequence, the small amplitude pulses did not change the state of the memristor.

### 3.2. Memristor Emulation Comparison Measurements

The following measurements were carried out on four different network types and on a single memristor for reference. The measuring signal is alternating write–erase sinusoidal pulses with a length of 23 ms in a sequence of 50 cycles.

This measurement is supposed to simulate a general training scenario, where an analogue memristor characteristic is expected and the training is done by several small pulses. According to this consideration, the writing pulses of the measurement have not enough energy to change the state of a single memristor into its ON state.

**Figure 6:**
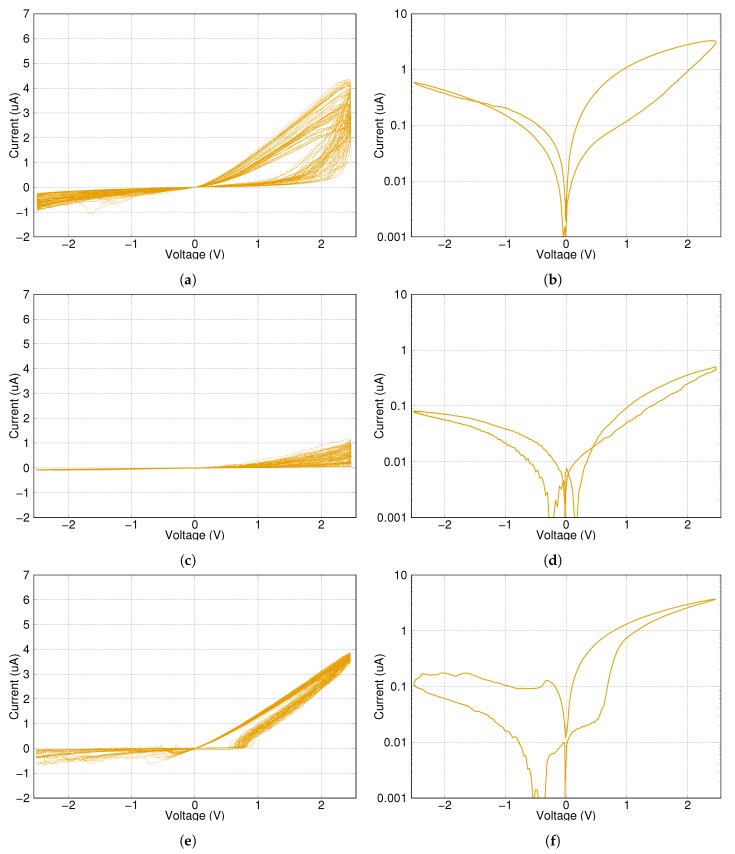
Short-time pulses on a single memristor, the checkerboard like and the H-fractal memristor network, respectively. (**a**,**c**) and (**e**) show the voltage–current diagram of the whole signal. (**b**,**d**,**f**) are the average of all 50 write–erase signals on a logarithmic scale.

**Figure 7:**
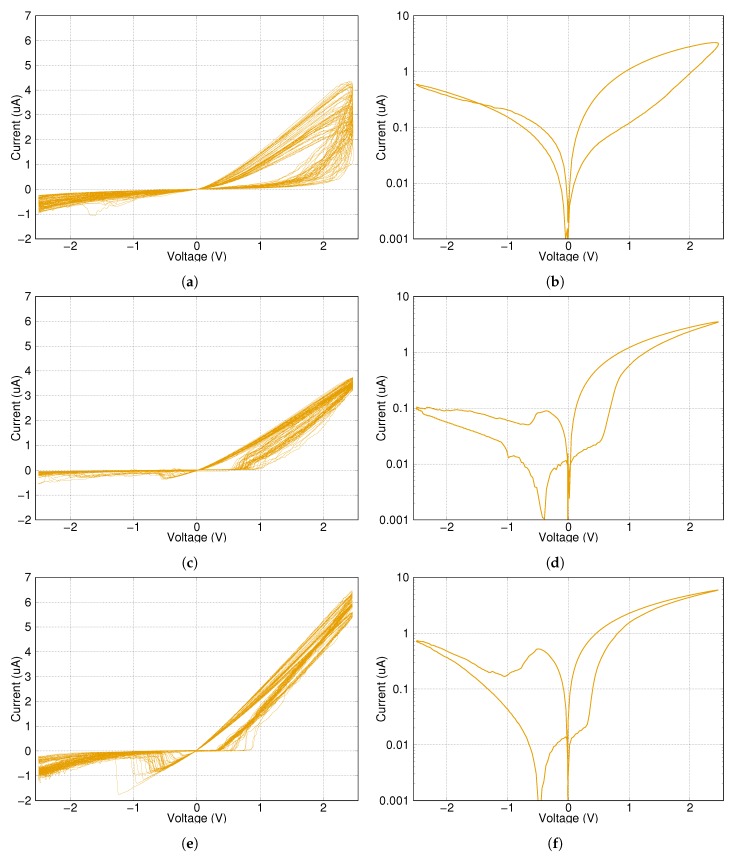
Short-time pulses on a single memristor, the new three dimensional network with sixteen memristors and the reduced network with twelve memristors, respectively. (**a**,**c**) and (**e**) show the voltage–current diagram of the whole signal. (**b**,**d**) and (**f**) are the average of all 50 write–erase signals on a logarithmic scale.

The results can be seen in Figure 6 and Figure 7. Subfigures (**a**),(**c**) and (**e**) show the voltage–current diagram of the whole signal. Subfigures (**b**),(**d**) and (**f**) are the voltage–current diagrams of the average of all 50 write–erase signals with the current shown on a logarithmic scale.

## 4. Discussion

The checkerboard type of network was practically unable to switch its state significantly compared to other solutions. This was probably due to the limited number of parallel connections in the network, which produced less possible routes to open. Higher control voltage could change its state, but the risk of device damage increases with the increased after-switch current. Longer pulses could also help, but it makes the writing process slower.

The results for the H-fractal type of network are very similar to the newly introduced network regarding the writing of the state into low resistance position. However, this type of network has problems with erasing the state into the OFF state and could get stuck at an in-between state.

The ON and OFF resistance values of the network with twelve memristors are lower than the other networks due to the reduced number of serial layers. However, when one compares it to a single memristor, it has lower ON resistance value and higher OFF resistance, meaning the network is more sensitive to control signals than only one memristor. In other words, a pulse with the same voltage level could make a clearer distinction between the initial and after states.

The previous simulation results suggested that the switching speed could decrease using memristor grids. Surprisingly, the switching speed did not decrease, but increased instead. The networks are approximately three times faster than a single memristor. This is fairly unexpected, as the control voltage stayed constant in both measurements, which means that the voltage on any single memristor in a network measurement had to be strictly lower than in the case of a single device measurement at any given time during measuring.

One explanation of this phenomenon could be the following: under the threshold voltage, the device behaves as a very small capacitor. As the metal flows into the dielectric matter to build up the filament, the partially charged capacitor discharges, causing a short-time high-energy electric current burst. The other devices are sensitive to fast current changes and the filament forming is starting in them as well. It can be seen as a “domino effect” with the consecutive memristors. If any of the OFF state memristors in a series switches to the ON state, the rest will automatically switch as well immediately after.

If any of the memristors which closes the source in the series, opens, the rest will automatically open immediately after.

Based on the above presented measurements the following parameter values were acquired, presented in Table 1. The resistance values are the average ON/OFF ratio values of the 50 cycle long measurement sequence.

Another important feature of th networks to note is the stronger nanobattery effect [16]. This causes the visible shift of the zero current level after the erasing pulse. The nanobattery effect is undesired in most applications, but can be dealt with by an appropriate control voltage and timing. It can also be taken advantage of, in some scenarios.

## 5. Conclusions

Two new types of memristor networks have been introduced, which are able to emulate more reliable memristors. Measurements have been successfully carried out for both the previously presented networks and the new networks. The measurements provided new information about the macro-characteristics of memristor networks compared to the previous simulations. The increased switching speed of memristor networks should be further investigated. This solution can be used with existing devices to support the implementation of neuromorphic applications.

## Figures and Tables

**Table 1:** The table shows the main properties of emulating memristor networks. Higher ON/OFF ratio is considered better and the best values are indicated accordingly, namely the highest OFF resistance, the lowest ON resistance and the highest overall ON/OFF ratio. Lower dispersion is also considered better. The lowest is indicated.

Measured Object	OFF Resistance	ON Resistance	ON/OFF Ratio	Dispersion Index
Single memristor	5.7889 MΩ	0.7185 MΩ	8.0569	0.4553
H-fractal network	19.472 MΩ	0.6717 MΩ	28.990	0.02718
Checkerboard network	20.322 MΩ	5.4633 MΩ	3.7197	0.04921
3D 2 × 2 × 4 network	20.651MΩ	0.7072 MΩ	29.201	0.01800
3D 2 × 2 × 3 network	9.3426 MΩ	0.4194MΩ	22.276	0.02491

## Abbreviations

The following abbreviations are used in this manuscript:

VLSI	Very large scale integration
APM	Analog purpose memristor
DPM	Digital (or discrete) purpose memristor
BPM	Binary purpose memristor
CMOS	Complementary metal–oxide–semiconductor
RAM	Random access memory
FPGA	Field programmable gate array
